# Gut microbiota-associated metabolite trimethylamine *N*-Oxide and the risk of stroke: a systematic review and dose–response meta-analysis

**DOI:** 10.1186/s12937-020-00592-2

**Published:** 2020-07-30

**Authors:** Mahdieh Abbasalizad Farhangi, Mahdi Vajdi, Mohammad Asghari-Jafarabadi

**Affiliations:** 1 Research Center for Evidence Based Medicine, Health Management and Safety Promotion Research Institute, Tabriz University of Medical Sciences, Attar Neyshabouri, Daneshgah Blv, Tabriz, Iran; 2 Drug Applied Research Center, Tabriz University of Medical Sciences, Tabriz, Iran; 3 Road Traffic Injury Research Center, Department of Epidemiology and Biostatistics, Faculty of Health, Tabriz University of Medical Sciences, Tabriz, Iran

**Keywords:** Stroke, Trimethylamine *N*-oxide (TMAO), Observational studies, Dose-response analysis, Gut microbiota metabolite, Risk factor

## Abstract

**Aims:**

Several epidemiological studies have examined the association between trimethylamine *N*-Oxide (TMAO) and stroke risk; however, the results are still inconclusive. The purpose of this meta-analysis was to evaluate the relationship between TMAO concentrations and stroke risk.

**Methods:**

PubMed, Scopus, Cochrane and ProQuest search engines were systematically searched up to 18 June 2019. All of the studies that evaluated the relationship between TMAO and stroke were included in the systematic review and eligible studies were included into the meta-analysis. Meta-regression and subgroup analysis were also employed to find the source of heterogeneity.

**Results:**

Eight studies (two cross-sectional studies, two cohort studies, three case-control studies and one nested case-control study) with a total of 6150 participants were included in the meta-analysis. The overall result showed that being in the highest category of TMAO increased the odds of stroke by 68% (OR: 1.675; CI: 0.866–3.243; *P* = 0.047) and mean TMAO concentrations was 2.201 μmol/L higher in patients with stroke rather than non-stroke controls (weighted mean difference (WMD): 2.20; CI: 1.213–3.188; *P* < 0.001). Furthermore, we observed revealed a non-linear association between increased TMAO levels and increased odds of stroke (P- for nonlinearity < 0.001). In addition, visual inspection of the funnel plot revealed a significant asymmetry among studies examining the differences in TMAO in patients with stroke versus control group.

**Conclusion:**

This is the first meta-analysis to show positive dose-dependent relations between circulating TMAO concentration and stroke risk. However, further interventional studies and long-term studies are needed to better explain causality.

## Introduction

Prevalence of stroke and stroke- related deaths has increased dramatically in most countries [1]. Every year, stroke happens to 15 million people around the world and is currently the second most important cause of death worldwide [2]. Because traditional risk factors do not fully account for stroke risk, the identification of novel stroke risk factors may lead to additional means to improve stroke prevention and decrease burden of disease [3, 4]. Recent studies in human subjects have revealed a close relation between the gut microbiota and several diseases such as cardiovascular disease (CVD), atherosclerosis and stroke [5–10].

The gut microbiota is the collection of microorganisms such as bacteria, archaea, eukarya and viruses that exists in the human gastrointestinal tract creating a diverse ecosystem [11]. The homeostasis of the gut microbiota is important for preserving human health and wellness [10]. Some dietary nutrients such as L-carnitine, phosphatidylcholine and choline are especially processed by intestinal bacteria to produce trimethylamine (TMA), which is absorbed by the intestine and converted into trimethylamine-*N*-Oxide (TMAO) in the liver by flavin-containing monooxygenase 3 [12–14]. It has been demonstrated that there is a positive association between high TMAO concentration and adverse health outcomes such as obesity, inflammation**,** cardiovascular events and total mortality [9, 15–19]. Several studies have proposed a possible relationship between TMAO and stroke risk, but their results are controversial [7, 20, 21]. For example, several studies revealed reduced TMAO level and some other found no significant relationship between stroke and TMAO levels; in a case control study by Yin J et al. [15], a significant dysbiosis of the gut microbiota has been revealed in stroke patients; however, plasma TMAO concentration in patients with stroke and transient ischemic attack (TIA) patients was lower than control patients with asymptomatic atherosclerosis. Similarly, in other study, there was a gradual decrease in the risk of stroke by increasing TMAO concentrations [22]. However, several studies have revealed that the TMAO concentrations is higher in the ischemic and hemorrhagic stroke and its higher concentration is in parallel of the stoke severity [7, 20]. In 2019, Stubbs JR et al. [21] reported that highest versus lowest quintile of TMAO was related to higher prevalence of stroke (11% versus 9%). In another study by Rexidamu M et al. [20] increased serum TMAO concentrations in patients with stroke compared with controls (*P* < 0.001) was reported; moreover, the odds of severe stroke compared with mild stroke in association of increased serum TMAO levels was 1.22 (CI: 1.08–1.32; *P* < 0.001). Nie et al. [7] reported that higher TMAO concentration was related to an increased risk of a first stroke. Subjects in the highest category of TMAO had 34% higher risk of first stroke compared to subjects in the lowest category. Differences in disease status and characteristics of participants across studies might affect TMAO concentrations [23, 24] and consequently partially explain such discrepancy. The possible underlying mechanisms for the positive association between risk of stroke at higher TMAO concentrations are involvement of TMAO in atherosclerotic lesions, increased foam cell production by TMAO-mediated increased expression of scavenger receptors on macrophages, changes in bile acid metabolism, cholesterol, sterol and up-regulation of pro-inflammatory pathways [6].

From the clinical point of view, because of the challenges in the risk stratification of stroke, biomarkers could provide clinically useful information regarding the disease scoring algorithm. The attention in combining biochemical and clinical markers for use in accuracy medicine is consequently growing and metabolomics is a comparatively new and promising technology for identifying valuable biomarkers [6, 25]. Clearly, these discrepancies warrant a need for a summative study for elucidation of the real association between TMAO and stroke. To our knowledge, no study has comprehensively examined the relationship between TMAO and stroke risk. Therefore, we carried out a meta-analysis of all relevant, eligible published studies to evaluate the association between gut-microbe-derived TMAO and risk of stroke.

## Methods and materials

### Study protocol

The Preferred Reporting Items of Systematic Reviews and Meta-Analysis (PRISMA) guideline was followed to perform this review (Supplementary Table 1) [26]. The 12-item PRISMA extension checklist was also followed to write the Abstract [27]. The protocol of this study was registered in the International prospective register of systematic reviews (PROSPERO) with the protocol number CRD42019143010.

### Search strategy

A systematic literature search was conducted from inception to June 2019 in Scopus, PubMed, ProQuest and Cochrane to identify studies investigating the association between gut-microbe-derived TMAO and risk of stroke. Two authors (MAF and MV) conducted the literature search independently. Then the results were compared and discussed until the authors reached an agreement. The search was limited to only studies published in English.

The following medical subject headings (MESH) were used in the literature search: (TMAO OR trimethylamine n-oxide) AND (stroke OR prestroke OR poststroke OR hypertension OR blood pressure OR serum lipids OR total cholesterol OR insulin resistance OR cardiovascular disease OR CVD OR diabetes OR metabolic syndrome OR obesity). Since “stroke” as our main study outcome was included as secondary or tertiary findings in several manuscripts and was not stated in the title or abstract, we included all of the relevant key words in our search key terms for to ensure we captured all relevant papers. Meanwhile, the reference lists of the selected studies and relevant review studies were also manually checked for additional data sources.

### Study selection

The articles were considered to be eligible if they met the following criteria: 1) the study design was observational (case-control, analytic cross-sectional, nested case-control, case-cohort or cohort); 2) reported the outcomes according to TMAO categories for at least two categories for performing two class mean-difference meta-analysis; 3) reported the outcomes according to TMAO categories for at least three categories for performing dose response meta-analysis of prevalence or risk estimates; 4) reported the results according to TMAO categories for at least three categories for performing dose-response meta-analysis of continuous variables; 5) reported the outcome of interest as the mean and standard deviation (SD) of continuous variables 6) reported hazard ratios (HRs), relative risks (RRs) or odds ratios (ORs) and the corresponding 95% confidence intervals (CI) of stroke incidence for the highest versus the lowest levels of TMAO; 7) reported the number of cases and participants/person-years in highest versus the lowest levels of TMAO, or reported sufficient information to allow estimation of those numbers. In the case of cohort studies, we only included the baseline characteristics in our analysis and information after follow-up was not included. In addition, we excluded interventional studies, in vitro studies, animal studies, review articles, letters, case reports, and studies that were carried out among pregnant or lactating women and children.

### Quality assessment and data extraction

The quality assessments of the studies were carried out by two independent reviewers (MAF, MV) and any disagreements were resolved by consensus opinion. We used the Newcastle–Ottawa Scale (NOS) [28] to evaluate the quality of the case-control and cohort studies, and we used Agency for Healthcare Research and Quality (AHRQ) guidelines to evaluate the quality of cross-sectional studies [29]. Two investigators (MAF, MV) independently extracted data from included studies by using a standard extraction form. Data extracted included first author’s name, country of origin, publication year, study design, participants’ age and sex, median or mean TMAO range, number of participants in highest versus the lowest levels of TMAO, source of TMAO (plasma or serum), the reported mean (SD) or risk estimates and the 95% CIs of study results among categories of TMAO.

### Statistical analysis

All statistical analyses were performed using STATA version 13.0 (STATA Corp, College Station, TX), and *P*-value < 0.05 was considered as statistically significant.

### Two-class meta-analysis of the comparison of mean TMAO in patients with and without stroke or the prevalence of stroke in highest versus lowest TMAO categories

In two class meta-analysis, the studies that reported the mean TMAO levels in patients with and without stroke were included. The mean and SD of variable (TMAO) was used to estimate the unstandardized mean differences as the effect size calculated by pooled estimate of weighted mean difference (WMD) with 95% confidence interval (CI) in the case and control group. When the mean values were missed and median and range were provided [7, 20] we used the method provided by Hozo et al. [30] considering the median values as best estimate of mean for sample size more than 25 and calculating SD as follows: ${S}^2\approx \left(\frac{1}{12}\right(\frac{{\left(a-2m+b\right)}^2}{4}+{\left(b-a\right)}^2$). When the number of participants in each category of TMAO was not provided in the study, we assumed that equal number of subjects is enrolled in each group. The pooled stroke prevalence was compared between highest versus lowest categories of TMAO concentrations. ORs and 95% CIs were applied to estimate the combined effects.

The Z-test was used to estimate the overall effect size, and *p* < 0.05 (2-tailed) was considered statistically significant. When there was significant heterogeneity (e.g. more than 50%) in the fixed effect model, a random effect model was applied to conduct meta-analyses. The heterogeneity between studies was evaluated by I^2^ index and Cochrane’s Q test [31]. The heterogeneity was deemed significant if either the I^2^ > 50% or Q statistic had *p* < 0.1. Subgroup analyses were done by study location, quality, the prevalence of diabetes, sample source, design, gender, hypertension, age and smoking status. Publication bias was assessed visually by Begg’s funnel plots and statistically with Begg’s test and Egger’s test.

### Dose response meta-analysis of prevalence of stroke

For dose response meta-analysis, only the studies that reported at least three TMAO categories and the prevalence of stroke were included. The included articles were categorized based on disease status, the design of the studies and the blood sample type of TMAO measurement [7, 13, 21, 22, 32]. The linear dose-response association was estimated using a two-stage generalized least-squares trend estimation, as earlier described [33]. Also, we identify the median point in each TMAO category.

If medians were not presented in the study, we estimated the midpoint of the upper and lowerlimits in each category as the median. When the highest category was open-ended, its TMAO level was estimated by assuming that the interval was the same as the closest category. The lowest category of TMAO concentration was used as the reference dose for each study. Any potential non- linear relations were explored using random-effects dose-response meta-analysis by defining the restricted cubic splines with three knots at fixed percentiles (10, 50 and 90%) of distribution [34] and these information were used to estimate study-specific OR estimates per 1 μmol/ L of TMAO increments.

## Results

### Literature search and study characteristics

**Fig. 1:**
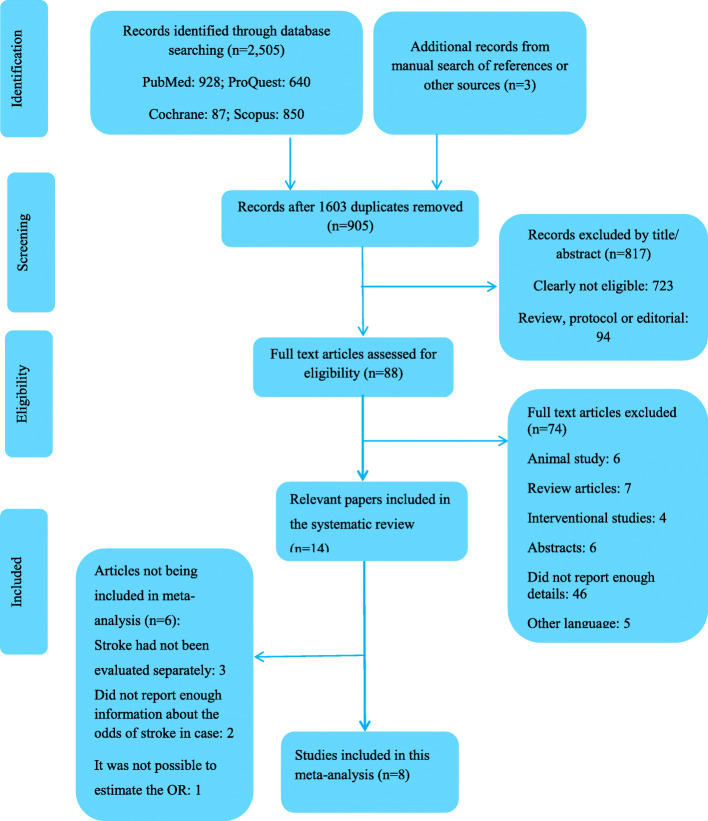
Flow diagram of study screening and selection process. The search of database and other sources identified 905 potentially relevant studies after duplicate exclusion. In addition, 897 studies were excluded after further evaluations. Finally, 8 studies were selected for this meta-analysis

In total, 2505 studies were retrieved in our initial search and three studies were identified by manual search. After removing 1603 duplicates and screening the titles and abstracts, 88 full-texts of potentially relevant studies were retained for further evaluation. We excluded 74 articles due to the following reasons: seven studies were review, five studies were not English language, 46 studies had insufficient data, six studies were performed on animals, six studies were abstract only and 4 studies were interventional studies. Finally, 14 papers were included in this systematic review and meta-analysis. The flowchart of study selection is presented in Fig. 1.

The general characteristics of the included studies in the current review are mentioned in the Table 1. Overall, six studies were cohort [9, 21, 32, 37–39], two were cross-sectional [13, 21], two were nested-case control [7, 16] and four were case-control studies [15, 20, 22, 36]. The sample size in the included studies ranged from 78 to 4007. In total, 14,660 individuals were participated in these studies and all of them were assessed both genders without separate analysis. Five studies were performed in the China [7, 9, 15, 20, 36], four in USA [22, 32, 38, 39] and remaining studies were performed in Japan [13], North Korea [16], Germany [37], Denmark [35] and one trial involving 22 countries [21]. In nine studies plasma TMAO was evaluated [9, 15, 22, 32, 35–39] while in five studies serum TMAO was investigated [7, 13, 16, 20, 21]. In the included studies, higher TMAO levels were related to higher risk of major cardiovascular events including stroke or higher TMAO concentrations were reported in the patients with stroke versus non-stroke patients. From the included studies in the systematic review (Table 1), three reported higher risk (either OR, RR or HR) of stroke in the highest TMAO categories [7, 20, 21], two reported higher prevalence of stroke in highest versus lowest category of TMAO levels [21, 32], five reported higher serum or plasma TMAO concentrations in highest versus lowest category of TMAO levels [7, 15, 20, 36, 38] and one reported the higher incidence of new ischemic brain lesions in highest versus lowest TMAO categories (OR: 3.85 (1.37–7.56; *P* < 0.001) [9]. Three studies also reported the positive associations between higher serum or plasma TMAO levels and adverse cardiac outcomes including stroke [16, 37, 39]. Three studies reported no significant relationship between odds of stroke and serum TMAO or no significant difference in the prevalence of stroke in highest versus lowest TMAO categories [13, 22, 35]. From those studies, in four studies [16, 37–39], stroke was included as one of the major adverse cardiovascular outcomes and had not been evaluated separately. Consequently, these studies were excluded from the meta-analysis. One study had information of hazard ratio (HR) of stroke occurrence after median of 15 years follow-up. Therefore, it was not possible to estimate the OR [35] and another study [9] provided the odds of new brain lesions incidence in patients with or without previous lesions and did not provide the information about the odds of stroke in case group versus control group [9]. These six studies were not included in the meta-analysis. The results of the quality assessment indicated that the NOS score ranged from 7 to 8 and 5 to 9 for the case-control and cohort studies, respectively and the quality score of the AHRQ methodology checklist ranged from 7 to 8 for cross-sectional studies [13, 21] (Supplementary Table 2–4).

**Table 1:** The characteristics of the studies included in the systematic review of the association between TMAO and stroke

first author	Year/ country	Disease status	Total Num. of participants	Num. of categories/ num. Each group	Design	Sample source	TMAO μmol/ lit	Age range (y)	Male %	Main Results	Adjustments
Zheng L et al. [16]	2019/ North Korea	Community based general population	192	4/86	Nested case-control	Serum TMAO	CVD: 1.57 (0.79–2.29) μmol/L versus Control: 0.68 (0.23–1.40) μmol/L	≥ 35	35.41	The odds of CVD (defined as CHD+ stroke) at highest TMAO quartile was significantly higher than the lowest (OR 2.73 CI: 1.32–5.63)	SBP, BMI, use of anti-HTN, smoking, drinking, T2DM, TC, TG, HDL-C, eGFR
Winther SA et al. [35]	2019/ Denmark	Type1 Diabetes	1159	4/ 290	Cohort/ median 15 years follow-up	Plasma TMAO	5.7 (3.8–9.9)	46 ± 13	58%	The HR of relation between incident stroke and TMAO was 1.08 (0.93–1.27) *P* = 0.33	age, sex, DM duration, HbA1c, SBP, TC, smoking, UAER
Stubbs JR et al. [21]	2019/ Baseline data of EVOLVE trial of 22 countries	Patients receiving maintenance hemodialysis	1243	5/ 248	Cross-sectional	Serum TMAO	2.5–1103.1	54 ± 14 (50–60)	60%	Higher prevalence of stroke in highest (11%) versus lowest (9%) TMAO quintiles; the HR/SHR of the plasma TMAO and stroke was OR:1.20 (CI: 0.88 to 1.64)	age, sex, BMI, SBP, albumin, race, dialysis-duration, smoking, CVD, history of coronary intervention, stroke, MI, BUN
Rexidamu M et al. [20]	2019/ China	Patients with first acute ischemic stroke	510	2/ 255	Case- control	Serum TMAO	Mean: 0.5–18.3 μM, Median: 5.8 (IQR: 3.3–10.0)	65 (IQR: 57–71)	53.3	Mean serum TMAO in patients stroke was higher than controls (*P* < 0.001). The odds of severe stroke with TMAO levels was 1.22 CI:1.08–1.32) (*P* < 0.001)	Age, CRP, HCY and other factors
Liang Z et al. [36]	2018/ China	Patients with arterial fibrillation	179	2 (68/111)	Case-control	Plasma TMAO	Stroke versus non-stroke (8.25 ± 1.58 μM versus 2.22 ± 0.09)	Stroke versus non stroke (68.0 ± 9.6; 64.1 ± 13.3)	58.10	Significantly higher plasma TMAO in stroke versus non-stroke; the odds ratio of association between TMAO and stroke was 4.934 (*P* < 0.001)	–
Wu C et al. [9]	2018/ China	Patient’s with CAS	268	2 (117/ 151)	Cohort / 30 day follow up for developing new lesions	Plasma TMAO	New lesions versus non-new lesions median 5.2 versus 3.2 μmol/L	64.4	56.7	Higher risk of new ischemic brain lesions in highest versus lowest TMAO quartiles (OR: 3.85 (1.37–7.56) (*P* < 0.001)	Age, sex, symptomatic CAS%, CAS, SBP, FSG, LDL-C, HDL-C, hcys, % aortic arch III
Nie J et al. [7]	2018/ China	Incident stroke and matched control, using data from the CSPPT	1244	2/ 622	Nested case-control	Serum TMAO	Stroke: 2.5 (1.6–4.0) control: 2.3 (1.4–3.7)	(45–75)	47%	Higher serum TMAO in patients with stroke compared with controls (2.5 versus 2.3 μmol/L) and higher odds of stroke in highest versus lowest TMAO tertile (OR:1.43 (1.02–2.01) *P* = 0.04	SBP, BMI, FSG, TC, eGFR, hcys, folate, smoking, time-averaged SBP in treatment period, choline, L carnitine
Haghikia A et al. [37]	2018/ Germany	Patients with incident stroke	78	4/20	Cohort / 1 year follow-up	Plasma TMAO	–	59 ± 14	69%	Higher odds of incident CVD event (including stroke) in highest versus lowest TMAO quartile OR: 2.31; 95% CI, 1.25–4.23; *P* < 0.01	Age, sex, HTN, T2DM, LDL-C, smoking
Haghikia A et al. [37]	2018/ Germany	Patients with incident stroke	593	4/148	Cohort / 1 year follow-up	Plasma TMAO	–	67 ± 13	61%	Higher odds of incident CVD event (including stroke) in highest versus lowest TMAO quartile OR: 3.3; 95% CI, 1.2–10.9; P = 0.04)	age, sex, HTN, T2DM, LDL, smoking
Tang WHW et al. [32]	2017/ USA	Patients with T2DM	1216	3 /401	Cohort / 5 years follow-up	Plasma TMAO	4.4 (2.8–7.7)	64.4 ± 10.2	58%	Significantly higher prevalence of stroke history in highest versus lowest TMAO tertiles (12% versus 5%; *P* = 0.002). Increased odds of major adverse cardiac risk including stroke in highest versus lowest TMAO tertiels (OR: 1.94 (1.23–3.05) P < 0.001)	Age, gender, history of CVD, history of HF, SBP, LDL-C, HDL-C, smoking, BMI, hsCRP, HbA_1_C, eGFR.
Li X et al. [38]	2017/ USA	Patinets with CVD (Cleveland acute coronary syndrome cohort)	530	2 (220/ 310)	Cohort /7 years follow-up	Plasma TMAO	4.28 (2.55–7.91)	62.4 ± 13.9	57.5	Higher plasma TMAO in patients with adverse cardiac events (including stroke) compared without (5.09 versus 3.73); P < 0.001	Age, gender, HDL-C, LDL-C, smoking, history of DM, HTN, CAD, CRP, eGFR, troponin T, STEMI, NSTEMI or unstable angina
Li X et al. [38]	2017/ USA	Patients with CVD (Swiss ACS cohort)	1683	2 (190/ 1493)	Cohort/ 7 years follow-up	Plasma TMAO	2.87 (1.94–4.85)	63.9 ± 12.4	77.8	Higher plasma TMAO in patients with adverse cardiac events (including stroke) compared without (3.75 versus 2.80); P < 0.001	Age, gender, HDL-C, LDL-C, smoking, history of DM, HTN, revas-cularization or CAD, CRP, eGFR, troponin T, STEMI, NSTEMI or unstable angina
Guasch-Ferre M et al. [22]	2017/ USA	Patients with CVD	980	4/ 245	Case-cohort	Plasma TMAO	–	55–80	46.12	No significant association between HR of stroke in TMAO tertiels (*P* = 0.31)	Age, sex, family history of CVD, smoking, BMI, PA, HTN, T2DM
Mafune A et al. [13]	2016/ Japan	Patients underwent CVD surgeries	227	4/ 56–57	Cross-sectional	Serum TMAO	0.09 to 141.2	68	70	No significant difference in prevalence of stroke between quartiles of TMAO (*P* = 0.49)	–
Yin J et al. [15]	2015/ China	Patients with ischemic or TIA stroke	551	2 (322/ 231)	Case- control	Plasma TMAO	Stroke versus controls (2.70; 1.91)	18–80	63.70	Plasma TMAO was lower in patients with stroke compared with controls (P < 0.001)	–
Tang WHW et al. [39]	2013/ USA	Patients underwent CABG	4007	2 (513/3494)	Cohort/ 3 years follow-up	Plasma TMAO	3.7 (2.4–6.2)	63	64	Plasma TMAO was significantly higher in patients with adverse events (including stroke) compared with controls (P < 0.001); increased odds of events in forth quartiles versus first (1.43 (1.05–1.94))	Age, sex, smoking status, SBP, LDL-C, HDL-C, DM, hs-CRP, myeloperoxidase level, eGFR, WBC-count, BMI, medications (aspirin, statin, ACE inhibitor, ARB, or beta-blocker, extent of disease

Abbreviations: *ACEI* Angiotensin converting enzyme inhibitor, ACS *Acute coronary syndromes*, *ARB* Angiotensin receptor blockers, *BMI* Body mass index, *BUN* Blood urea nitrogen, *CABG* Coronary artery bypass surgery, *CAD Coronary artery disease*, *CAS* Carotid artery stenosis, *CI* Confidence interval*, CRP* C-reactive protein, *CSPPT* China Stroke Primary Prevention Trial, *CVD* Cardiovascular disease, *DM* Diabetes mellitus, *e-GFR* Estimated glomerular filtration rate, *EVOLVE* valuation of Cinacalcet Hydrochloride Therapy to Lower Cardiovascular Events*, FSG* Fasting serum glucose, *HbA1c* Hemoglobin A1C, *HCY* Homocysteine, *HDL-C* High density lipoprotein cholesterol, *HF* Heart failure, *HR* Hazard ratio, *HTN* Hypertension, *IQR* Interquartile range, *LDL-C* Low density lipoprotein cholesterol, *MI* Myocardial infarction, *NSTEMI* non–ST-segment elevation myocardial infarction*, OR* Odds ratio, *PA* Physical Activity*, SBP* Systolic Blood Pressure, *SHR* Subdistribution Hazard Ratio, *STEMI* ST-Elevation Myocardial Infarction, *TC* Total cholesterol, *T*_*2*_*DM* Type two diabetes, *TG* Triglyceride, TIA transient ischemic attack, *TMAO* Trimethylamine *N*-oxide, *UAER* urinary albumin excretion, *USA* United States, *WBC* White blood cells

### Finding from the two-class meta-analysis

**Fig. 2:**
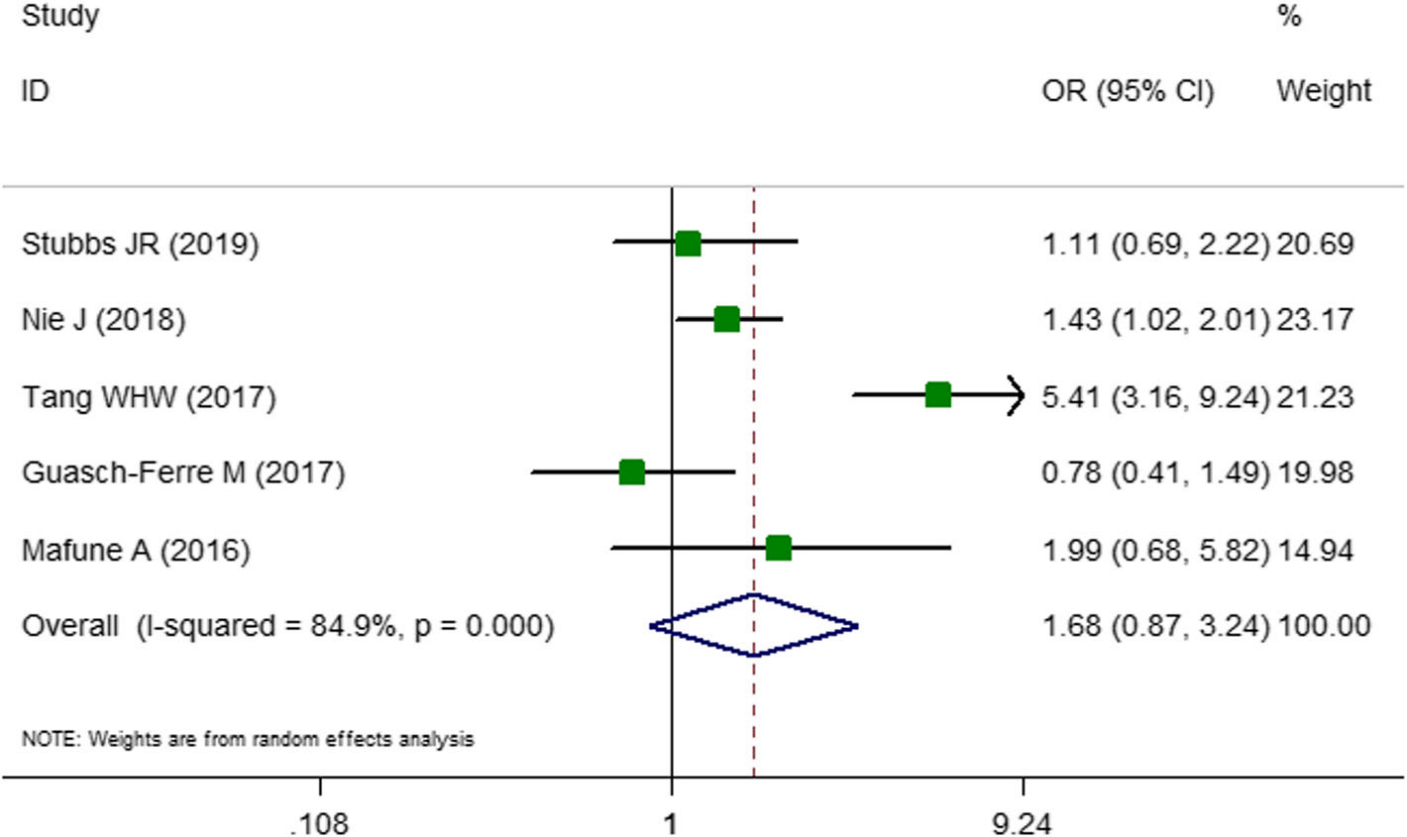
Forest plot presenting odds ratio (OR) for stroke in the highest versus the lowest category of trimethylamine *N*-oxide (TMAO) concentrations. As shown in Fig. 2, five studies were included in the analysis of TMAO concentrations and risk of stroke. The result showed that being in the highest category of TMAO is associated with 68% increased odds of stroke (OR: 1.67, 95% confidence interval (CI): 0.86–3.24, *P* = 0.04), with a significant heterogeneity (I^2^ = 84.9, *P* < 0.001). I^2^ represents the degree of heterogeneity

**Fig. 3:**
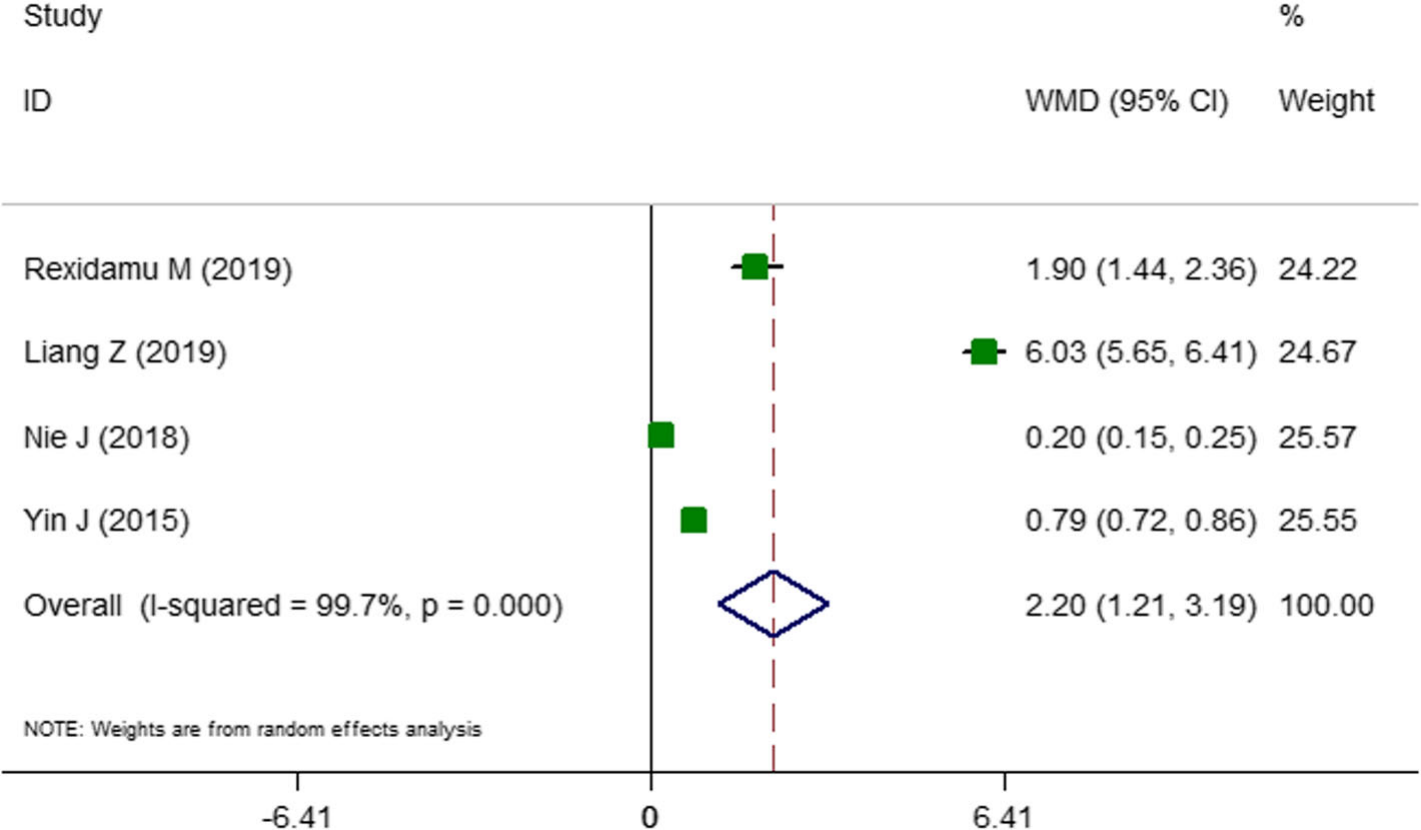
Forest plots of weighted mean difference (WMD) with 95% confidence interval (CI) for the circulating levels of trimethylamine *N*-oxide (TMAO) in stroke versus non-stroke patients. As shown in Fig. 3, four studies were included in the analysis. The result showed that mean TMAO concentrations was 2.20 μmol/L higher in patients with stroke compared with non-stroke controls (WMD: 2.20, 95% CI: 1.21–3.18, *P* < 0.001), with a significant heterogeneity (I^2^ = 99.7%, P < 0.001). I^2^ represents the degree of heterogeneity

The first two-class meta-analysis was performed for identifying the association between odds ratio of the stroke and high TMAO concentration and the findings are presented in Fig. 2. In total, five studies [7, 13, 21, 22, 32] with 4910 subjects were included in two-class meta-analysis. Two of these studies were cross-sectional studies [13, 21] and three [7, 22, 32] were cohort studies or were extracted from cohort studies (case-cohort or nested case-control studies). These articles reported either the prevalence or the odds of stroke in the highest versus lowest **category of** TMAO levels according to the baseline characteristics of participants. In the fixed-effect model, a high heterogeneity value was observed (OR: 1.65; CI: 1.31–2.09; *P* < 0.001; I^2^ = 84.9%; P _heterogeneity_ < 0.001; data not shown). Therefore, the random effect model was performed and found that being in the highest category of TMAO is related to 68% increased odds of stroke (OR: 1.67; CI: 0.86–3.24; *P* = 0.04; I^2^ = 84.9; P _heterogeneity_ < 0.001; Fig. 2). The results of subgrouping for stroke risk are presented in Tables 2. Subgroup analyses to explore heterogeneity presented that the level of heterogeneity was reduced in subgrouping according to age, sample source, disease status, location and the proportion of diabetes. The reported data about the medication use was not sufficient enough to include into subgrouping. The second two-class meta-analysis was conducted to the comparison of circulating TMAO in patients with and without stroke. In total, four studies [7, 15, 20, 36] with the total number of 2484 of participants were included. All these studies were performed in both genders and three studies were designed as case-control studies [15, 20, 36] and one was nested case-control study [7]. In the fixed-effect model, a high heterogeneity score was obtained (standardized mean difference (SMD): 0.54; CI: 0.46–0.62; *P* < 0.001; I^2^ = 98.8% data not shown). Therefore, the random effect model was performed with the corresponding forest plots (Fig. 3). Accordingly, mean TMAO concentrations was 2.20 μmol/L higher in patients with stroke compared with non-stroke controls (WMD: 2.20; CI: 1.21–3.18; P < 0.001, I^2^ = 99.7%; Fig. 3). Subgrouping did not reveal any potential source of heterogeneity (Table 3). All of the studies were carried out in China and were recruited among patients with stroke. Therefore, the subgrouping according to disease status and geographical location was not performed.

**Table 2:** Subgroup analyses for the prevalence of stroke in highest versus lowest trimethylamine *N*-oxide (TMAO) categories

Group	No. of studies	WMD (95% CI)	P _**within group**_	P _between group*_	Effect model	P _**heterogeneity**_	I^**2**^, %
**Total**	5	1.67 (0.86, 3.24)	0.047		Random	< 0.001	84.9
**Location**							
USA	2	2.07 (0.31, 13.82)	0.451	0.056	Random	< 0.001	95.1
Asia	2	1.47 (1.06, 2.03)	0.019		Fixed	0.565	0
EVOLVE trial	1	1.11 (0.61, 1.99)	0.721				–
**Study quality**				0.004			
High	3	2.31 (0.76, 7.02)	0.137		Random	< 0.001	87.1
Moderate	2	1.12 (0.63, 2.01)	0.687		Random	0.103	62.4
**Design**							
*Cross-sectional*	2	1.26 (0.75, 2.11)	0.36	< 0.001	Fixed	0.349	0
*Cohort*	1	5.41 (3.16, 9.25)	< 0.001		Fixed	–	–
*Case-cohort*	1	0.78 (0.40, 1.48)	0.45		Fixed	–	–
*Nested case-control*	1	1.43 (1.01, 2.00)	0.039		Fixed	–	–
**Sample**				0.024			
*Plasma*	2	2.07 (0.31, 13.82)	0.452		Random	< 0.001	95.1
*Serum*	3	1.37 (1.03, 1.83)	0.026		Fixed	0.6	0.0
**Disease status**				< 0.001			
*Patients with CVD*	2	1.12 (0.45, 2.75)	0.797		Random	0.143	53.5
*Stroke*	1	1.43 (1.01, 2.00)	0.039		Fixed	0	0
*Renal disorders*	1	1.11 (0.61, 1.99)	0.721		Fixed	0	0
*T*_*2*_*DM*	1	5.41 (3.16, 9.25)	< 0.001		Fixed	0	0
**Sample size**				0.048			
*1000 <*	3	2.03 (0.83, 4.95)	0.116		Random	< 0.001	90.3
*≥ 1000*	2	1.12 (0.45, 2.75)	0.797		Random	0.143	53.5
**Mean/ median age (y)**				0.023			
≥ 65	2	1.12 (0.45, 2.75)	0.726		Fixed	–	0
60–65	2	2.73 (0.74, 10.07)	0..130		Random	< 0.001	94.1
< 60	1	1.11 (0.61, 1.99)	0.797		Random	0.143	53.5
**Male %**				0.004			
< 55	2	1.12 (0.63, 2.01)	0.687			0.103	62.4
≥ 55	3	2.31 (0.76, 7.02)	0.137			< 0.001	87.1
**HTN%**				< 0.001			
< 80	2	3.67 (1.41, 9.54)	0.008		Random	0.102	62.5
≥ 80	3	1.17 (0.83, 1.64)	0.356		Fixed	0.248	28.3
**Diabetes %**				0.024			
< 50	3	1.37 (1.03, 1.83)	0.026		Fixed	0.6	0
≥ 50	2	2.45 (1.62, 3.70)	< 0.001		Random	< 0.001	95.1
**Current smoking %**				< 0.001			
< 40	3	1.17 (0.83, 1.64)	0.356		Fixed	0.248	28.3
≥ 40	2	3.67 (1.41, 9.54)	0.008		Random	0.102	62.5

Abbreviations: *EVOLVE* valuation of Cinacalcet Hydrochloride Therapy to Lower Cardiovascular Events, *CI* Confidence interval, CVD cardiovascular disease, *T*_*2*_*DM* Type two diabetes, *HTN* Hypertension, *WMD* Weighted mean difference *Between groups comparisons are obtained from inverse variance method

**Table 3:** Subgroup analyses for the association between trimethylamine *N*-oxide (TMAO) and stroke

Group	No. of studies	WMD (95% CI)	P _**within group**_	P _between group*_	Effect model	P _**heterogeneity**_	I^**2**^, %
**Total**	4	2.20 (1.21, 3.18)	< 0.001	< 0.001	Random	< 0.001	99.7
**Study quality**							
High	1	0.45 (0.34, 0.57)	< 0.001		Fixed	–	–
Moderate	3	2.82 (1.19, 4.46)	0.001		Random	< 0.001	99.2
**Design**				< 0.001			
*Case- control*	3	2.90 (−0.39, 6.20)	0.085		Random	< 0.001	99.7
*Nested case-control*	1	0.20 (0.15, 0.24)	< 0.001				–
**Sample**				< 0.001			
*Plasma*	2	0.49 (−0.08, 1.07)	0.094		Random	< 0.001	99.4
*Serum*	2	3.96 (−0.08, 8.01)	0.05		Random	< 0.001	99.5
***Sample size***				< 0.001			
*< 550*	2	3.96 (−0.08, 8.01)	0.055		Random	< 0.001	99.5
*≥ 550*	2	0.49 (− 0.08, 1.07)	0.094		Random	< 0.001	99.4
**Mean/ median age (y)**				< 0.001			
< 60	1	0.45 (0.34, 0.57)	< 0.001		Fixed	–	–
60–65	3	2.82 (1.19, 4.46)	0.001		Random	< 0.001	99.2
**% Male**				< 0.001			
*< 55*	2	1.03 (−0.63, 2.69)	0.224		Random	< 0.001	98.1
*≥ 55*	2	3.40 (−1.72, 8.54)	0.194		Random	< 0.001	99.9
**HTN%**				< 0.001			
< 70	1	6.18 (5.47, 6.89)	< 0.001		Fixed	–	–
≥ 70	2	0.57 (0.32, 0.82)	< 0.001		Random	0.018	82.2
N/A	1	1.77 (1.57, 1.97)	< 0.001		Fixed	–	–
**Diabetes %**				< 0.001			
< 20	2	3.31 (−2.30, 8.92)	0.248		Random	< 0.001	99.6
≥ 20	2	1.24 (0.20, 2.28)	0.019		Random	< 0.001	98.4
**Current smoking %**				< 0.001			
< 30	2	0.57 (0.32, 0.82)	< 0.001		Random	0.018	82.2
≥ 30	1	6.18 (5.47, 6.89)	< 0.001		Fixed	–	–
N/A	1	1.77 (1.57, 1.97)	< 0.001		Fixed	–	–

Abbreviations: *CI* Confidence interval, *HTN* Hypertension, *WMD* Weighted mean difference *Between groups comparisons are obtained from inverse variance method

### Findings from the dose-response meta-analysis

**Fig. 4:**
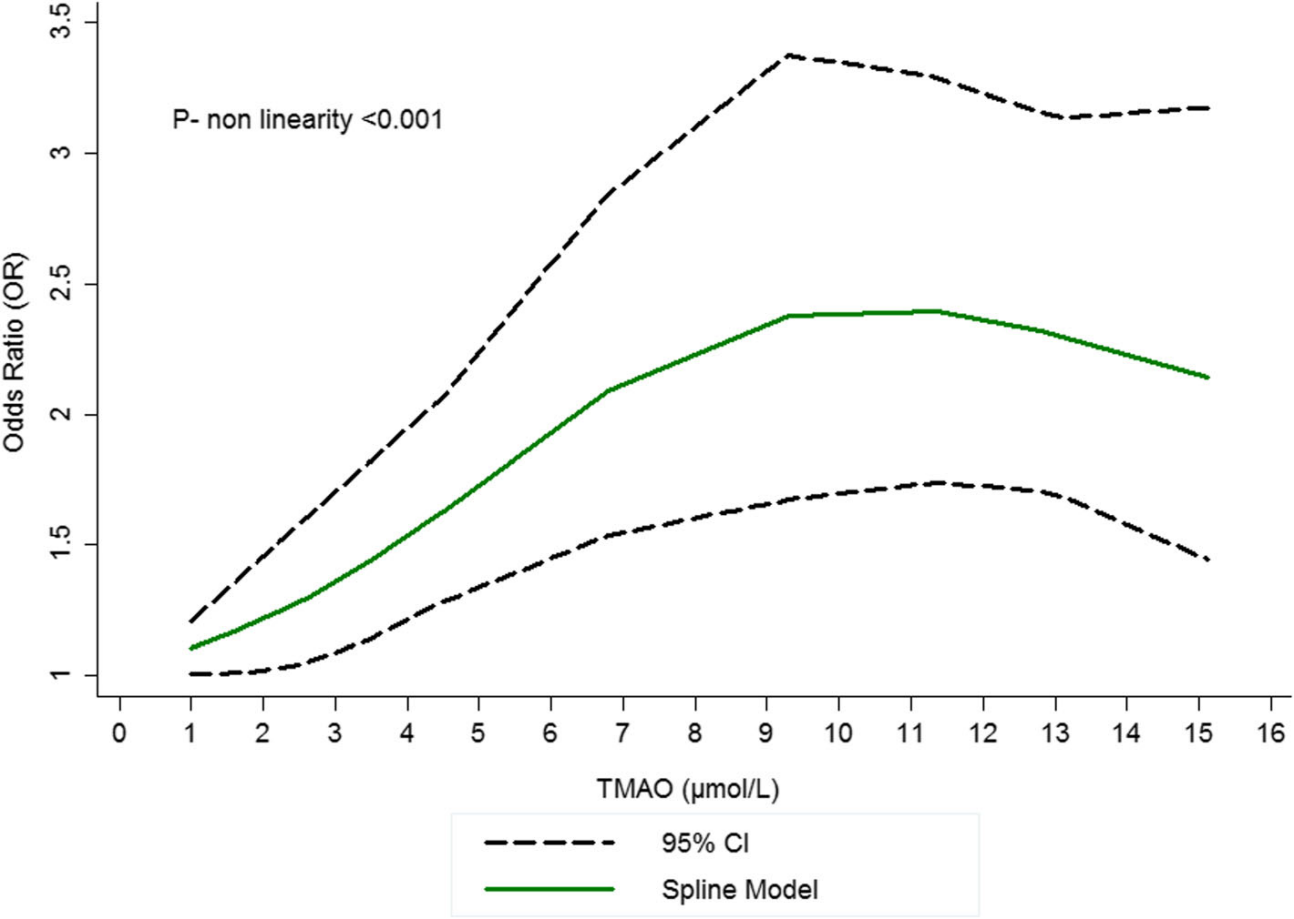
Dose–response association between the circulating trimethylamine *N*-oxide (TMAO) concentration and stroke risk. Linear relation (solid line) and 95% confidence interval (CI) (dashed lines) of pooled odds ratio (OR) of stroke prevalence by 1 μmol/L increment of circulating TMAO. As shown in Fig. 4, there is a sharp increase in the related curve of stroke risk between the dosages of ~ 0–10 μmol/L of circulating TMAO and after that dose, a slight reduction in risk of stroke is observed

In total, five studies [7, 13, 21, 22, 32] with the total of 4910 participants were included in the dose-response meta-analysis; there was an evidence of non-linear relationship between high TMAO levels and increased odds of stroke as presented by significant *P* values of less than 0.05 (*n* = 5 studies; P- for nonlinearity < 0.001; Fig. 4). The results revealed a pooled OR of 1.05 (95% CI: 1.03, 1.08) per 1-μmol/L increment in TMAO concentrations, OR of 1.03 (95% CI: 1.02, 1.04) per 5 *μ* mol/L increment in TMAO concentrations and OR of 1.078 (95% CI: 1.06, 1.09) per 10-*μ*mol/L increment in TMAO concentrations demonstrating that the risk of stroke increases by 3 and 8% for 5 and 10 *μ* mol/L increments in TMAO concentrations. As shown in Fig. 4, there is a sharp increase in the related curve of stroke risk between the dosages of ~ 0–10 μmol/L of circulating TMAO and after that dose, a slight reduction in risk of stroke is observed.

### Publication bias

The funnel plots are shown in Supplementary Fig. 1A and B. Visual inspection of the funnel plot revealed a significant asymmetry among studies examining the differences in TMAO in patients with stroke versus control group, suggesting the existence of bias, while no evidence of asymmetry was found among studies examining the effect of TMAO on odds of stroke. Furthermore, no evidence of publication bias was found by the Egger’s test and Begg’s test for prevalence of stroke in highest versus lowest TMAO categories [e.g. Begg test (*P* = 0.327) and Egger test (*P* = 0.197)] or circulating TMAO concentration in patients with and without stroke [e.g. Begg test (*P* = 0.734) and Egger test (*P* = 0.170)].

## Discussion

In the current systematic review and meta-analysis, we summarized available records from 14 observational studies which examined the relationship between circulating TMAO concentrations and stroke risk in adults. In the current study, increased TMAO levels were associated with increased risk of stroke either in a two-class or dose-response manner as a summary of existing evidence.

The present meta-analysis provided supportive results that are in-line with previous narrative review, which identified that TMAO is a risk factor of CVD and stroke [6]. Also, these results are partly in agreement with the findings from prior meta-analyses, which identified that elevated serum concentrations of TMAO were independent risk factors for major adverse cardiovascular events and mortality [19, 40]. While most studies focus on CVD, some studies include cerebrovascular events in their outcomes. A growing body of evidence suggests the increased risk of stroke and major cardiovascular events in higher TMAO levels [21, 36], however, several inconsistencies in the relationship between circulating TMAO and stroke risk are a matter of debate [15, 16, 22, 35]. For instance, Guasch-Ferre et al. [22] failed to show any significant association between TMAO and risk of stroke. A similar result was found by Mafune et al. [13] in 227 patients undergoing elective coronary angiography.

It seems that the possible explanation for these discrepancies is the dose-dependent relationship between TMAO and stroke; as revealed in our meta-analysis, the relationship between high TMAO concentrations and the risk of stroke is non-linear dose-dependent association (P- for nonlinearity < 0.001) and there is a sharp increase in the risk of stroke between the dosages of ~ 0–10 μmol/L of circulating TMAO and after that dose, a slight reduction in risk of stroke is observed. Interestingly, in our previous meta-analysis this dose-dependent association was also observed between high TMAO concentrations and body weight in healthy individuals and this increase was only observed in dosage of ~ 0–10 μmol/L of circulating TMAO [17]. These results further highlight the dose-dependent relationship between TMAO and risk factors. Although because of the low number of included studies making a relevant scientific judgment is not possible. The studies included in our dose-response meta-analysis were conducted in different disease (e.g. hemodialysis, CVD, T2DM and stroke), sample type (plasma and serum) and race (White and Black) and therefore it was not possible to control the possible confounding effect of the disease. Moreover, in the current meta-analysis, patients with stroke had 2.20 μmol/L higher circulating TMAO concentrations compared with non-stroke controls. Our findings offer an explanation for the prior conflicting evidence on TMAO levels in stroke patients. While most of the studies reported higher TMAO concentrations, one study found lower TMAO concentrations in stroke patients [15]. Yin et al. [15] found that patients with different severities of stroke had different diversity of gut microbiota, which may explain the inconsistency in different studies. Also, Shafi et al. [41] found that TMAO had a linear increase in risk with cardiovascular mortality in white hemodialysis patients, but not in black patients, proposing the relationship between TMAO and results might differ by ethnic and race groups. Disease status might affect the association between TMAO and stroke. In the present meta-analysis, diabetes was identified as one the heterogeneity sources. Several studies reported that diabetes is associated with variations in gut microbiota [42, 43]. In a meta-analysis by Zhuang et al. [44], the authors found a dose-dependent relationship between TMAO and risk of diabetes. It is possible that microbiota variation due to diabetes might regulate the circulating TMAO levels and accordingly contribute to the heterogeneity in our study.

The low number of studies regarding the relationship between TMAO and stroke made as unable to perform subgrouping according to baseline TMAO concentrations and disease status and conducting further studies in this field are needed. However, for two-class meta-analysis we performed subgrouping because of the high level of between- study heterogeneity. We assumed that this high heterogeneity is one of the possible major challenges to clarify the relationship between stroke and circulating TMAO levels. In this meta-analysis, subgroup analysis shown that geographical location, sample source, age, disease status and the proportion of diabetes explained this heterogeneity. The effect of geographical location observed in our study has been confirmed in previous book written by Cittadini A et al. [45]. The role of geographical location in the heterogeneity could also be possibly due to the role of dietary habits and diet in regulation of circulating TMAO levels [46–48] and several previous studies have demonstrated the efficacy of different dietary patterns (e.g western dietary habits, Mediterranean dietary patterns) on gut microbiota [46, 47, 49]. Furthermore, recent studies have reported that diet not only affects the gut microbiota but also affects the levels of TMAO in blood [50, 51]. Consumption of milk, egg yolk, organ and muscle meats and fish is known to increase the urine and blood levels of TMAO [52, 53].

The underlying mechanism behind the relationship between elevated TMAO levels and increased stroke risk is still not fully clarified. A possible explanation is that TMAO may involve in the prolongation of angiotensin effects, which is also likely to worsen cardiac remodeling and may lead to harmful effect in heart failure [54]. Also, TMAO may induce platelet aggregation by the stimulation of cytoplasmic calcium release, by which it may predispose to a hyper-coagulating status and increased thrombotic events [55]. A significant proportion of ischemic stroke is mainly due to large-artery atherosclerosis, whereas TMAO accelerates atherosclerosis with increased macrophage cholesterol accumulation and foam cell formation [56]; stimulated vascular inflammation [57] and endothelial dysfunction [58]. Also, several studies have revealed a positive relationship between TMAO and cardio-metabolic stroke-related risk factors such as impaired glucose metabolism, insulin resistance and metabolic syndrome [59, 60]. These effects are important mechanisms of ischemic stroke. The investigators propose that the suppression of TMA creation or reducing plasma levels of TMAO can serve as a way of inhibiting diet induced atherosclerosis [61, 62].

Because long-term monitoring of circulating TMAO levels in patients before they develop stroke is absent in existing studies, it is generally difficult to decide whether a high TMAO level is a causing factor for stroke. The findings of the current meta-analysis have important clinical implications highlighting the role of TMAO in stroke incidence and developing preventive or therapeutic approaches to reduce its concentrations. More prospective cohort studies are required to confirm a causal association; also, interventional studies could help to determine the impact of modulation of TMAO levels as a new therapeutic target for stroke. Although several interventional studies exist that investigated the effects of several dietary interventions like carnitine [63, 64], plant based- diets [65, 66], probiotics [67, 68] on TMAO concentrations, however, because of conflicting data about the role of diet in circulating TMAO (e.g. the higher production of TMAO after fish consumption [69]) it will be difficult to clarify the exact association between diet and TMAO levels. Moreover, it has been demonstrated that gut microbiota and intra-individual variations in the population of several families of bacteria belonged to the Proteobacteria phyla and Firmicutes in the human intestine could be an important determinant of the association between TMAO and the disease [70, 71]; In a study by Manor et al. [72], gut microbiota activity was one of the most important determinants of TMAO particularly in individuals with higher kidney function. To the best of our knowledge, this is the first dose-response meta-analysis that suggests a positive association between high TMAO level and stroke risk. In this review, we included only observational studies and followed the PRISMA statement. Also, for two-class meta-analysis we performed subgrouping because of the high level of between- study heterogeneity. However, our review has some limitations. First of all, no causal relationship between TMAO levels and stroke risk can be inferred from our meta-analysis of observational studies; second, articles that did not provide adequate information for pooling were excluded from the meta-analysis, which may increase risk of bias in the total effects by TMAO. Third, several potential confounders such as the genetic variations and dietary patterns might also affect the results. Potential sources of heterogeneity were assessed using different methods and there was a high heterogeneity in the present meta-analysis with the possible recognized sources of sample source, location, age, and disease status. Finally, this dose-response meta-analysis could only reveal the potential temporal relationship between increased circulating TMAO and subsequent stroke risk. Whether increased TMAO was causative to stroke warrants interventional studies.

## Conclusions

In the present meta-analysis we showed the positive relationship between circulating TMAO and stroke in dose-response and two-class meta-analyses. Moreover, patients with stroke had higher circulating TMAO concentrations compared with non-stroke controls. Overall, increased TMAO levels increase the risk of stroke, while further interventional studies and long-term studies are needed to better explain causality.

## Supplementary information

**Additional file 1: Table S1.** PRISMA Checklist. **Table S2.** Newcastle-Ottawa Quality Assessment Scale (NOS) for cohort studies included in the systematic review and meta-analysis of the association between TMAO and stroke risk. **Table S3.** Newcastle-Ottawa Quality Assessment Scale (NOS) for case-control studies included in the systematic review and meta-analysis of the association between TMAO and stroke risk **Table S4.** Agency for Healthcare Research and Quality (AHRQ) checklist to assess quality of the cross-sectional studies included in the meta-analysis of the association between TMAO and stroke risk.

**Additional file 2: Figure S1.** Begg’s funnel plot (A) of Two-class meta-analysis; (B) of Meta-analysis of continuous variables.

## Data Availability

All of the data are available with reasonable request from the corresponding author.
