# Supplementary material for: Gut microbiota-associated metabolite trimethylamine N-Oxide and the risk of stroke: a systematic review and dose–response meta-analysis
Source: Nutr J. 2020 Jul 30;19:76. doi: 10.1186/s12937-020-00592-2 (PMC7393891; doi:10.1186/s12937-020-00592-2)
Supplement: Supplementary file 1 — Additional file 1: Table S1. PRISMA Checklist. Table S2. Newcastle-Ottawa Quality Assessment Scale (NOS) for cohort studies included in the systematic review and meta-analysis of the association between TMAO and stroke risk. Table S3. Newcastle-Ottawa Quality Assessment Scale (NOS) for case-control studies included in the systematic review and meta-analysis of the association between TMAO and stroke risk Table S4. Agency for Healthcare Research and Quality (AHRQ) checklist to assess quality of the cross-sectional studies included in the meta-analysis of the association between TMAO and stroke risk. [file 12937_2020_592_MOESM1_ESM.docx]

**Title: Gut Microbiota-derived Metabolite Trimethylamine *N*-Oxide and increased risk of stroke: A Systematic Review and Dose–Response Meta-analysis**

**Authors**: Mahdieh Abbasalizad Farhangi, Mahdi Vajdi, Mohammad Asghari-Jafarabadi

**Corresponding to**: Dr Mahdieh Abbasalizad Farhangi

Email: [abbasalizad_m@yahoo.com](mailto:abbasalizad_m@yahoo.com)

**Key words:** Stroke, dose-response meta-analysis, trimethylamine *N*-oxide (TMAO), gut microbiota metabolite, risk factor.

**Supplementary Material**

**Supplementary Table 1- PRISMA Checklist [1]**

| **Section/topic** | **#** | | | **Checklist item** | **Reported on page #** |
| --- | --- | --- | --- | --- | --- |
| **TITLE** | | | | | Page 1 |
| Title | 1 | | | Identify the report as a systematic review, meta-analysis, or both. | Page 1  Lines 2-3 |
| **ABSTRACT** | | | | | Page 2 |
| Structured summary | 2 | | | Provide a structured summary including, as applicable: background; objectives; data sources; study eligibility criteria, participants, and interventions; study appraisal and synthesis methods; results; limitations; conclusions and implications of key findings; systematic review registration number. | Page 2  Lines 28-44 |
| **INTRODUCTION** | | | | | Page 3, 4 |
| Rationale | 3 | | | Describe the rationale for the review in the context of what is already known. | Page 3  Lines 72-76 |
| Objectives | 4 | | | Provide an explicit statement of questions being addressed with reference to participants, interventions, comparisons, outcomes, and study design (PICOS). | Page 4  Line 77-78 |
| **METHODS** | | | | | Page 4 |
| Protocol and registration | 5 | Indicate if a review protocol exists, if and where it can be accessed (e.g., Web address), and, if available, provide registration information including registration number. | | | Page 4  Lines 79-82 |
| Eligibility criteria | 6 | Specify study characteristics (e.g., PICOS, length of follow-up) and report characteristics (e.g., years considered, language, publication status) used as criteria for eligibility, giving rationale. | | | Page 4, 5  Lines 97- 108 |
| Information sources | 7 | Describe all information sources (e.g., databases with dates of coverage, contact with study authors to identify additional studies) in the search and date last searched. | | | Page 4  Line 84-97 |
| Search | 8 | Present full electronic search strategy for at least one database, including any limits used, such that it could be repeated. | | | Page 4  Line 84-97 |
| Study selection | 9 | State the process for selecting studies (i.e., screening, eligibility, included in systematic review, and, if applicable, included in the meta-analysis). | | | Page 4, 5  Lines 97- 108 |
| Data collection process | 10 | Describe method of data extraction from reports (e.g., piloted forms, independently, in duplicate) and any processes for obtaining and confirming data from investigators. | | | Page 5  Lines109- 123 |
| Data items | 11 | List and define all variables for which data were sought (e.g., PICOS, funding sources) and any assumptions and simplifications made. | | | Page 4, 5  Lines 97- 108 |
| Risk of bias in individual studies | 12 | Describe methods used for assessing risk of bias of individual studies (including specification of whether this was done at the study or outcome level), and how this information is to be used in any data synthesis. | | | Page 6  Lines 142-147 |
| Summary measures | 13 | State the principal summary measures (e.g., risk ratio, difference in means). | | | Page 5-7  Lines 123-160 |
| Synthesis of results | 14 | Describe the methods of handling data and combining results of studies, if done, including measures of consistency (e.g., I^2^) for each meta-analysis. | | | Page 5-7  Lines 123-160 |
| Risk of bias across studies | 15 | | Specify any assessment of risk of bias that may affect the cumulative evidence (e.g., publication bias, selective reporting within studies). | | Page 5-7  Lines 123-160 |
| Additional analyses | 16 | | Describe methods of additional analyses (e.g., sensitivity or subgroup analyses, meta-regression), if done, indicating which were pre-specified. | | Page 5-7  Lines 123-160 |
| **RESULTS** | | | | | Page 7 |
| Study selection | 17 | | Give numbers of studies screened, assessed for eligibility, and included in the review, with reasons for exclusions at each stage, ideally with a flow diagram. | | Page 7, 8  Lines 162-197  Figure 1 |
| Study characteristics | 18 | | For each study, present characteristics for which data were extracted (e.g., study size, PICOS, follow-up period) and provide the citations. | | Table 1 |
| Risk of bias within studies | 19 | | Present data on risk of bias of each study and, if available, any outcome level assessment (see item 12). | | Table 2, 3 |
| Results of individual studies | 20 | | For all outcomes considered (benefits or harms), present, for each study: (a) simple summary data for each intervention group (b) effect estimates and confidence intervals, ideally with a forest plot. | | Figures 2,3 |
| Synthesis of results | 21 | | Present results of each meta-analysis done, including confidence intervals and measures of consistency. | | Page 7, 9  Lines 162- 225 |
| Risk of bias across studies | 22 | | Present results of any assessment of risk of bias across studies (see Item 15). | | Figure 4, 5 |
| Additional analysis | 23 | | Give results of additional analyses, if done (e.g., sensitivity or subgroup analyses, meta-regression [see Item 16]). | | Page 7, 9  Lines 162- 225 |
| **DISCUSSION** | | | | | 233 |
| Summary of evidence | 24 | | Summarize the main findings including the strength of evidence for each main outcome; consider their relevance to key groups (e.g., healthcare providers, users, and policy makers). | | Page 10  Line 223 |
| Limitations | 25 | | Discuss limitations at study and outcome level (e.g., risk of bias), and at review-level (e.g., incomplete retrieval of identified research, reporting bias). | | Page 11  Lines 269- 279 |
| Conclusions | 26 | | Provide a general interpretation of the results in the context of other evidence, and implications for future research. | | Page 12  Lines 280- 290 |
| **FUNDING** | | | | | Page 12  Lines 295 |
| Funding | 27 | | Describe sources of funding for the systematic review and other support (e.g., supply of data); role of funders for the systematic review. | | Page 12  Lines 296 |

*From:*  Moher D, Liberati A, Tetzlaff J, Altman DG, The PRISMA Group (2009). Preferred Reporting Items for Systematic Reviews and Meta-Analyses: The PRISMA Statement. PLoS Med 6(7): e1000097. doi:10.1371/journal.pmed1000097

For more information, visit: **www.prisma-statement.org**.

**Supplementary Table 2- Newcastle-Ottawa Quality Assessment Scale (NOS) for cohort studies included in the systematic review and meta-analysis of the association between TMAO and stroke risk**

|  | **Selection** | | | | **Comparability** | **Outcome** | | | **Final score** |
| --- | --- | --- | --- | --- | --- | --- | --- | --- | --- |
| **Authors (Year)** | **Representativeness of the exposed cohort** | **Selection of the non-exposed cohort** | **Ascertainment of exposure** | **Demonstration that outcome of interest was not present at start of study** | **Comparability of cohorts on the basis of the design or analysis** | **Assessment of outcome** | **Was follow-up long enough for outcomes to occur** | **Adequacy of follow up of cohorts** |  |
| **Winter SA 2019 [2]** | * | * | * | * | ** | * | * | * | **9** |
| **Haghikia A 2018 ^a^ [3]** | - | * | * | * | * | * | - | - | **5** |
| **Haghikia A 2018 ^b^ [3]** | * | * | * | * | * | * | * | * | **8** |
| **Wu C 2018 [4]** | * | * | * | * | * | * | * | - | **7** |
| **Tang WHW 2013 [5]** | * | * | * | * | * | * | * | * | **9** |
| **Li X 2017 ^c^ [6]** | * | * | * | * | ** | * | * | * | **9** |
| **Li X 2017 ^d^ [6]** | * | * | * | * | ** | * | * | * | **9** |
| **Guasch-Ferre M [7]** | * | - | * | * | * | * | * | - | **6** |
| **Tang WHW 2017 [8]** | * | * | * | * | ** | * | * | * | **9** |

**a, first pilot cohort; b, Prospective Cohort With Incident Stroke (PCWIS); c, Cleveland acute coronary syndrome cohort; d, Swiss ACS cohort study**

One star represents a score of 1, and a study can be awarded a maximum score of 9 in total. The items were scored “*”if the answer was “YES,” and “-” if the answer was “NO” or “UNCLEAR.” The final quality scores were as follows: low quality = 0–3; moderate quality=4–7; high quality = 8–11.

**Supplementary Table 3- Newcastle-Ottawa Quality Assessment Scale (NOS) for case-control studies included in the systematic review and meta-analysis of the association between TMAO and stroke risk**

|  | **Selection** | | | | **Comparability** | **Exposure** | | | **Final score** |
| --- | --- | --- | --- | --- | --- | --- | --- | --- | --- |
| **Authors（Year）** | **Is the case definition adequate?** | **Representativeness of the cases** | **Selection of Controls** | **Definition of Controls** | **Comparability of cases and controls on the basis of the design or analysis** | **Ascertainment of exposure** | **Same method of ascertainment for cases and controls** | **Non-Response rate** |  |
| **Rexidamu M 2019 [9]** | * | * | * | * | * | * | * | - | **7** |
| **Liang Z 2019 [10]** | * | * | * | * | * | * | * | - | **7** |
| **Zheng L 2019 [11]** | * | * | * | * | ** | * | * | - | **8** |
| **Nie J 2018 [12]** | * | * | * | * | ** | * | * | - | **8** |
| **Yin J 2015 [13]** | * | * | * | * | * | * | * | - | **7** |

One star represents a score of 1, and a study can be awarded a maximum score of 9 in total. The items were scored “*”if the answer was “YES,” and “-” if the answer was “NO” or “UNCLEAR.” The final quality scores were as follows: low quality = 0–3; moderate quality=4–7; high quality = 8–11.

.

**Supplementary Table 4- Agency for Healthcare Research and Quality (AHRQ) checklist to assess quality of the cross-sectional studies included in the meta-analysis of the association between TMAO and stroke risk**

| **ARHQ Methodology Checklist items for Cross-Sectional study** | **Stubbs JR 2019 [14]** | **Mafune A 2016 [15]** |
| --- | --- | --- |
| 1) Define the source of information (survey, record review) | 1 | 1 |
| 2) List inclusion and exclusion criteria for exposed and unexposed subjects (cases and controls) or refer to previous publications | 1 | 1 |
| 3) Indicate time period used for identifying patients | 0 | 1 |
| 4) Indicate whether or not subjects were consecutive if not population-based | 1 | 1 |
| 5) Indicate if evaluators of subjective components of study were masked to other aspects of the status of the participants | 1 | 0 |
| 6) Describe any assessments undertaken for quality assurance purposes (e.g., test/retest of primary outcome measurements) | 1 | 0 |
| 7) Explain any patient exclusions from analysis | 1 | 1 |
| 8) Describe how confounding was assessed and/or controlled. | 1 | 1 |
| 9) If applicable, explain how missing data were handled in the analysis | 1 | 0 |
| 10) Summarize patient response rates and completeness of data collection | 0 | 1 |
| 11) Clarify what follow-up, if any, was expected and the percentage of patients for which incomplete data or follow-up was obtained | 0 | 0 |
| **Final score** | **8** | **7** |

If the answer was “NO” or “UNCLEAR” the item was scored “0”; conversely, the item was scored “1”. The full score for the scale is 11 points. The final quality scores were as follows: 0 to 3 was considered as low quality; 4 to 7 as moderate quality; and 8 to 11 as high quality.

**References**

1. Moher D, Liberati A, Tetzlaff J, Altman DG. Preferred reporting items for systematic reviews and meta-analyses: the PRISMA statement. Ann Intern Med. 2009;151:264-269.

2. Winther SA, Ollgaard JC, Tofte N, Tarnow L, Wang Z, Ahluwalia TS, et al. Utility of plasma concentration of trimethylamine n-oxide in predicting cardiovascular and renal complications in individuals with type 1 diabetes. Diabetes Care. 2019;42:1512–20.

3. Haghikia A, Li XS, Liman TG, Bledau N, Schmidt D, Zimmermann F, et al. Gut microbiota–dependent trimethylamine N-oxide predicts risk of cardiovascular events in patients with stroke and is related to proinflammatory monocytes. Arterioscler Thromb Vasc Biol. 2018;38:2225–35.

4. Wu C, Li C, Zhao W, Xie N, Yan F, Lian Y, et al. Elevated trimethylamine N-oxide related to ischemic brain lesions after carotid artery stenting. Neurology. 2018;90:e1283–90.

5. Tang WW, Wang Z, Levison BS, Koeth RA, Britt EB, Fu X, et al. Intestinal microbial metabolism of phosphatidylcholine and cardiovascular risk. N Engl J Med. 2013;368:1575–84.

6. Li XS, Obeid S, Klingenberg R, Gencer B, Mach F, Raber L, et al. Gut microbiota-dependent trimethylamine N-oxide in acute coronary syndromes: a prognostic marker for incident cardiovascular events beyond traditional risk factors. Eur Heart J. 2017;38:814–24.

7. Guasch-Ferre M, Hu FB, Ruiz-Canela M, Bullo M, Toledo E, Wang DD, et al. Plasma Metabolites From Choline Pathway and Risk of Cardiovascular Disease in the PREDIMED (Prevention With Mediterranean Diet) Study. J Am Heart Assoc. 2017;6:e006524.

8. Tang WW, Wang Z, Li XS, Fan Y, Li DS, Wu Y, et al. Increased trimethylamine N-oxide portends high mortality risk independent of glycemic control in patients with type 2 diabetes mellitus. Clin Chem. 2017;63:297–306.

9. Rexidamu M, Li H, Jin H, Huang J. Serum levels of Trimethylamine-N-oxide in patients with ischemic stroke. Biosci Rep. 2019;39.

10. Liang Z, Dong Z, Guo M, Shen Z, Yin D, Hu S, et al. Trimethylamine N‐oxide as a risk marker for ischemic stroke in patients with atrial fibrillation. J Biochem Mol Toxicol. 2019;33:e22246.

11. Zheng L, Zheng J, Xie Y, Li Z, Guo X, Sun G, et al. Serum gut microbe-dependent trimethylamine N-oxide improves the prediction of future cardiovascular disease in a community-based general population. Atherosclerosis. 2019;280:126–31.

12. Nie J, Xie L, Zhao BX, Li Y, Qiu B, Zhu F, et al. Serum Trimethylamine N-Oxide Concentration Is Positively Associated With First Stroke in Hypertensive Patients. Stroke. 2018;49:2021–28.

13. Yin J, Liao SX, He Y, Wang S, Xia GH, Liu FT, et al: Dysbiosis of Gut Microbiota With Reduced Trimethylamine-N-Oxide Level in Patients With Large-Artery Atherosclerotic Stroke or Transient Ischemic Attack. J Am Heart Assoc. 2015;4:e002699.

14. Stubbs JR, Stedman MR, Liu S, Long J, Franchetti Y, West RE, et al. Trimethylamine N-Oxide and Cardiovascular Outcomes in Patients with ESKD Receiving Maintenance Hemodialysis. Clin J Am Soc Nephrol. 2019;14:261–7.

15. Mafune A, Iwamoto T, Tsutsumi Y, Nakashima A, Yamamoto I, Yokoyama K, et al. Associations among serum trimethylamine-N-oxide (TMAO) levels, kidney function and infarcted coronary artery number in patients undergoing cardiovascular surgery: a cross-sectional study. Clin Exp Nephrol. 2016;20:731–9.
