# Supplementary figures and images for: Gut microbiota-associated metabolite trimethylamine N-Oxide and the risk of stroke: a systematic review and dose–response meta-analysis
Source: Nutr J. 2020 Jul 30;19:76. doi: 10.1186/s12937-020-00592-2 (PMC7393891; doi:10.1186/s12937-020-00592-2)

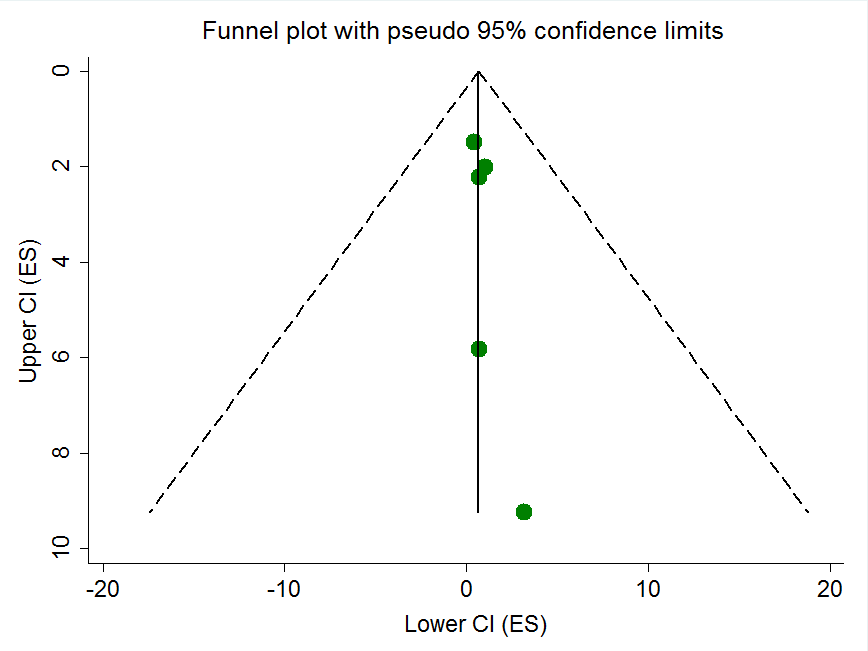

Supplement: Supplementary file 2 — Additional file 2: Figure S1. Begg’s funnel plot (A) of Two-class meta-analysis; (B) of Meta-analysis of continuous variables. [file 12937_2020_592_MOESM2_ESM.zip › Sup. Figure 1A. Funnel ORstrokeR5.tif]

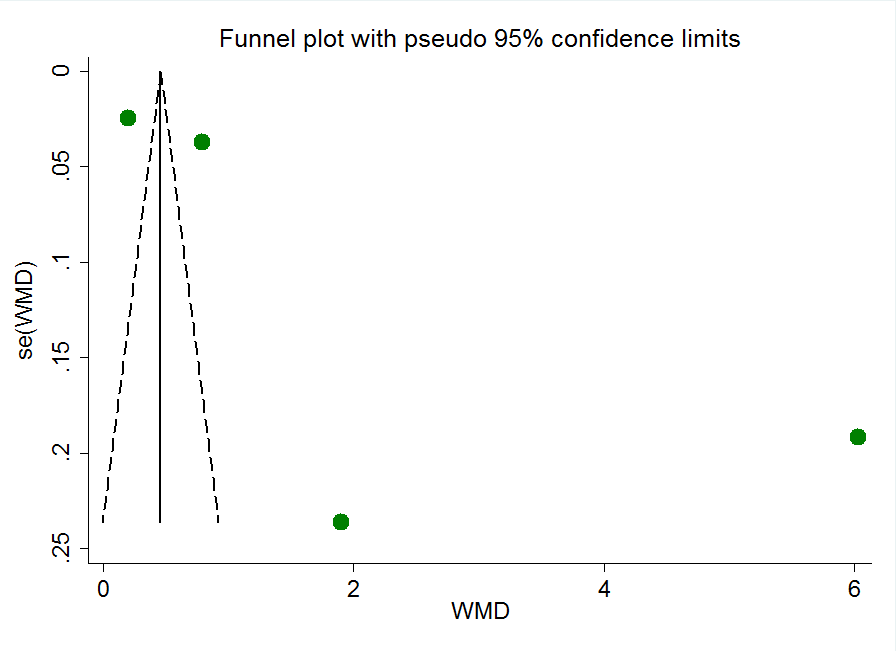

Supplement: Supplementary file 2 — Additional file 2: Figure S1. Begg’s funnel plot (A) of Two-class meta-analysis; (B) of Meta-analysis of continuous variables. [file 12937_2020_592_MOESM2_ESM.zip › sup. Figure 1B. Funnel MDstrokeR5.tif]
